# Impact of visual attention training on visual control and 3-point field goal percentage in semi-professional basketball players

**DOI:** 10.3389/fpsyg.2026.1567764

**Published:** 2026-02-17

**Authors:** Xiaokun Zhang, Wanting Li, Chunzhou Zhao

**Affiliations:** 1 College of Sports Science, Harbin Normal University, Harbin, China; 2 Harbin Sport University, Harbin, China; 3 College of P.E and Sports, Beijing Normal University, Beijing, China; 4 College of Physical Education, Chizhou University, Chizhou, China

**Keywords:** eye tracking, fixation behavior, shooting accuracy, sports performance, visual attention training

## Abstract

In the contemporary landscape of basketball, characterized by an escalating tempo and heightened defensive intensity, the 3-point shot has emerged as a pivotal weapon for teams striving to secure victory. Consequently, enhancing the 3-point field goal percentage has become an utmost concern for coaches and players alike. The objective of this study is to examine the influence of visual attention training on the visual attention characteristics and field goal percentage among semi-professional basketball players, thus establishing a scientific basis for basketball instruction and training. Twenty participants were randomly assigned to the experimental group and the control group based on their baseline 3-point field goal percentage (3PFGP) to ensure homogeneity between groups. The experimental group received an 8-week visual attention training protocol, whereas the control group adhered to routine training. A portable eye-tracking device was employed to collect eye movement metrics (e.g., number of fixations and durations) for both groups, with 3PFGP recorded pre-test, mid-test, and post-test. Data were analyzed using mixed ANOVA with Bonferroni-adjusted *post hoc* tests. Post-training analyses revealed significant differences in visual attention patterns and performance outcomes. Specifically, the experimental group exhibited reduced number of fixations on the hoop (Δ = −1.0), backboard (Δ = −1.2), and net (Δ = −0.8), alongside a prolonged fixation duration on the hoop (+115 ms, *p* < 0.05) and shortened durations on the backboard (−101 ms) and net (−202 ms, *p* < 0.01). Concurrently, a 5.0% improvement in 3PFGP was observed in the experimental group (*p* < 0.05), accompanied by a statistically significant redistribution of fixations across area of interest. These findings suggest that 8 weeks of visual attention training can enhance visual control ability during 3-point shooting, thereby improving shooting accuracy.

## Introduction

Basketball, as a globally prevalent team sport, demands sophisticated integration of physical prowess and cognitive strategies. Among critical performance determinants, 3-point shooting has emerged as a game-changing offensive weapon in modern basketball (Amaro et al., 2023), with its strategic value amplified by evolving gameplay dynamics characterized by accelerated tempo and intensified defensive schemes (Vencúrik et al., 2021; Marques et al., 2023). Three-point shooting efficiency offers a tangible scoring advantage over two-point attempts (Shea, 2020; Freitas, 2021), but requires greater biomechanical precision and cognitive control (Diotaiuti et al., 2021). Visual attention constitutes the neurocognitive substrate for superior shooting performance, governing both information acquisition (via fixation patterns) and action execution (through attentional focus modulation) (Conklin et al., 2020; Amaro et al., 2024).

The Quiet Eye (QE) framework posits that prolonged final fixation duration (≥600 ms) and reduced fixation transitions predict shooting success (Vickers, 1996; Oudejans et al., 2012). While QE is well-established, less is known about how structured training can systematically alter fixation patterns in semi-professional athletes and whether these changes translate to measurable performance gains in 3-point shooting (Giancamilli et al., 2022; Sagi, 2011). Neurocognitive models further identify external attentional focus as critical for performance optimization, enhancing movement automaticity through reduced conscious control (Wulf and Lewthwaite, 2016). Visual fixation serves as the primary modality through which participants acquire decision-making information, and the number of fixations reflects their individualized information search strategy (Causer et al., 2017; Zhao et al., 2023). The Constrained Action Hypothesis proposes that adopting an external focus enhances movement efficiency by promoting automated motor control (Matsumoto et al., 2022; Shooli et al., 2024). However, a synthesis of existing evidence reveals a gap in applying these principles to a structured, multi-week training regimen for 3-point shooting, particularly with semi-professional athletes.

Empirical evidence supports the malleability of attention. Visual fixation serves as the primary modality through which participants acquire decision-making information, and the number of fixations reflects their individualized information search strategy (Sirnik et al., 2022). The Constrained Action Hypothesis proposes that an external focus promotes automated motor control, minimizing conscious interference (Heusler and Sutter, 2022). In basketball, external focus training can improve free-throw performance by reducing excessive cognitive processing (Asadi et al., 2022; Lewthwaite and Wulf, 2017). Longer quiet eye durations and fewer fixations are associated with better shooting performance (Oudejans, 2005; Moeinirad et al., 2022). However, a synthesis of the literature reveals a gap: few studies have translated these principles into a multi-week, targeted training regimen for 3-point shooting with semi-professional athletes, whose skill level and training context differ from both novices and elites (Lu et al., 2020).

Therefore, the present study aimed to address this gap by designing and testing an 8-week visual attention training protocol grounded in Attentional Focus Theory and QE principles. We hypothesized that: (1) Compared to a control group, the experimental group would show a significant reduction in the number of fixations on the hoop, backboard, and net, and a significant increase in fixation duration on the hoop, following the training. (2) These optimized fixation behaviors would be associated with a significant improvement in 3-point field goal percentage in the experimental group.

## Materials and methods

### Participants

*A priori* power analysis was conducted using G*Power 3.1.9.7. For a repeated-measures ANOVA (within-between interaction) with two groups, three measurement points, an assumed medium effect size (*f* = 0.35, based on prior sports vision research), *α* = 0.05, and power (1−*β*) = 0.80, the required total sample size was 20 (Cohen, 1992; Lakens, 2014). Twenty female university basketball players were recruited. Participants were classified as semi-professional, defined as athletes competing at the national university level with structured, high-volume training, but not holding full-time professional contracts. They were selected from the women’s basketball teams of Beijing Normal University. Among them, 15 players were champions of the 2021 China University Women’s Basketball League, while five players participated in the 2023 World University Summer Games. These individuals had extensive competitive experience and a baseline 3-point field goal percentage exceeding 43%. Participants were randomly assigned to the experimental (*n* = 10) or control (*n* = 10) group using a computerized random number generator, stratified by their baseline 3PFGP to ensure group homogeneity. The age range was 20–25 years (*M* = 23.16, SD = 2.43). Mean height was 178.6 ± 5.2 cm, and mean body mass was 68.3 ± 7.1 kg. Each participant had over 10 years of experience (*M* = 11.12, SD = 1.54), and trained more than 15 h per week in the past year (*M* = 15.02, SD = 2.86). All participants provided written informed consent, were right-handed, and had normal or corrected-to-normal vision. The experimental protocol was approved by the Ethics Committee of the College of Physical Education and Sports at Beijing Normal University (No. 20221126). Pre-test measures of eye movement characteristics and 3PFGP did not differ between groups (*p* > 0.05).

### Design

The study employed a 3 (Time: pre-test, mid-test, post-test) × 2 (Group: experimental, control) mixed factorial design. The primary outcome variables were 3-point field goal percentage (3PFG%) and fixation duration on the hoop. Secondary outcomes included the number of fixations and fixation duration on the backboard and net. The independent variable was group assignment, and dependent variables were the eye movement indices and 3PFG%.

### Apparatus and calibration

The experiment employed the Tobii Glasses 3(Sweden), a state-of-the-art portable eye tracker featuring a sampling rate of 100 Hz and average accuracy of 0.03°. Before each testing session, a 5-point calibration procedure was performed for each participant to ensure accurate fixation mapping. The calibration was repeated if the average error exceeded 0.5°. This advanced device ensures minimal obstruction of the wearer’s field of view, enabling maximum freedom of head and body movement without sacrificing data accuracy (Zhao et al., 2024a). Our aim was to capture natural and authentic behavior to the greatest extent possible.

### Procedure

The study was conducted in a standardized indoor basketball arena from March 2 to April 30, 2024. The procedure consisted of three testing phases: pre-test (March 2–3), mid-test (April 1–2), and post-test (April 29–30). To minimize fatigue, testing was spread over 2 days per phase. Each day, participants completed a morning and an afternoon session. In each session, participants took 50 shots, organized into 10 blocks of 5 shots each, with a 3-min rest between blocks. This resulted in 100 shots per participant per testing phase. All shots were taken from the top of the key. Eye movement data were recorded during a separate dedicated testing session lasting approximately 120 min per phase, following the same shooting protocol. The training intervention occurred from March 6 to April 28.

### Experimental group training protocol

The 8-week intervention was grounded in Attentional Focus Theory (Wulf and Lewthwaite, 2016; Uludag et al., 2021). Aimed to cultivate expert-like fixation patterns through explicit instruction and feedback:

1. Attentional Focus Instruction: Before each shot, players were cued: “Focus your eyes on the front hoop of the hoop as soon as you catch the ball and keep them there until the ball is released.”
2. Feedback: Real-time verbal feedback was provided by the researcher during training if the player’s fixation shifted to the backboard or net prematurely. Feedback was phrased as, “Try to keep your eyes locked on the hoop longer.”
3. Progression: The first 2 weeks focused on form shooting with the cue. Weeks 3–6 incorporated catch-and-shoot drills. Weeks 7–8 added mild passive defensive pressure (a coach waving arms in peripheral vision).
4. Training Dose: Four 30-min sessions per week, with 100 shots per session (3,200 total shots). This volume was chosen to match the high-intensity, repetitive skill practice typical of elite university-level training programs and to ensure adequate dosage for neurocognitive adaptation. Injury risk was mitigated by integrating the drills into regular practice, ensuring proper warm-up, and monitoring player load.

### Control group training protocol

The control group performed traditional 3-point shooting practice for the same duration and volume (four 30-min sessions, 100 shots/session). They were organized into groups of three for rebounding and passing. Coaches provided technical feedback on shooting form (e.g., “bend your knees more,” “follow through”). To control for potential confounds, coaches were instructed not to give any cues related to fixation or visual attention. The primary distinction was the absence of the prescribed visual attention cues and feedback.

### Field goal percentage test

3PFG% testing was conducted concurrently with the eye movement test schedule on the same court. The procedure is described above.

### Division of area of interest (AOI)

**Figure 1:**
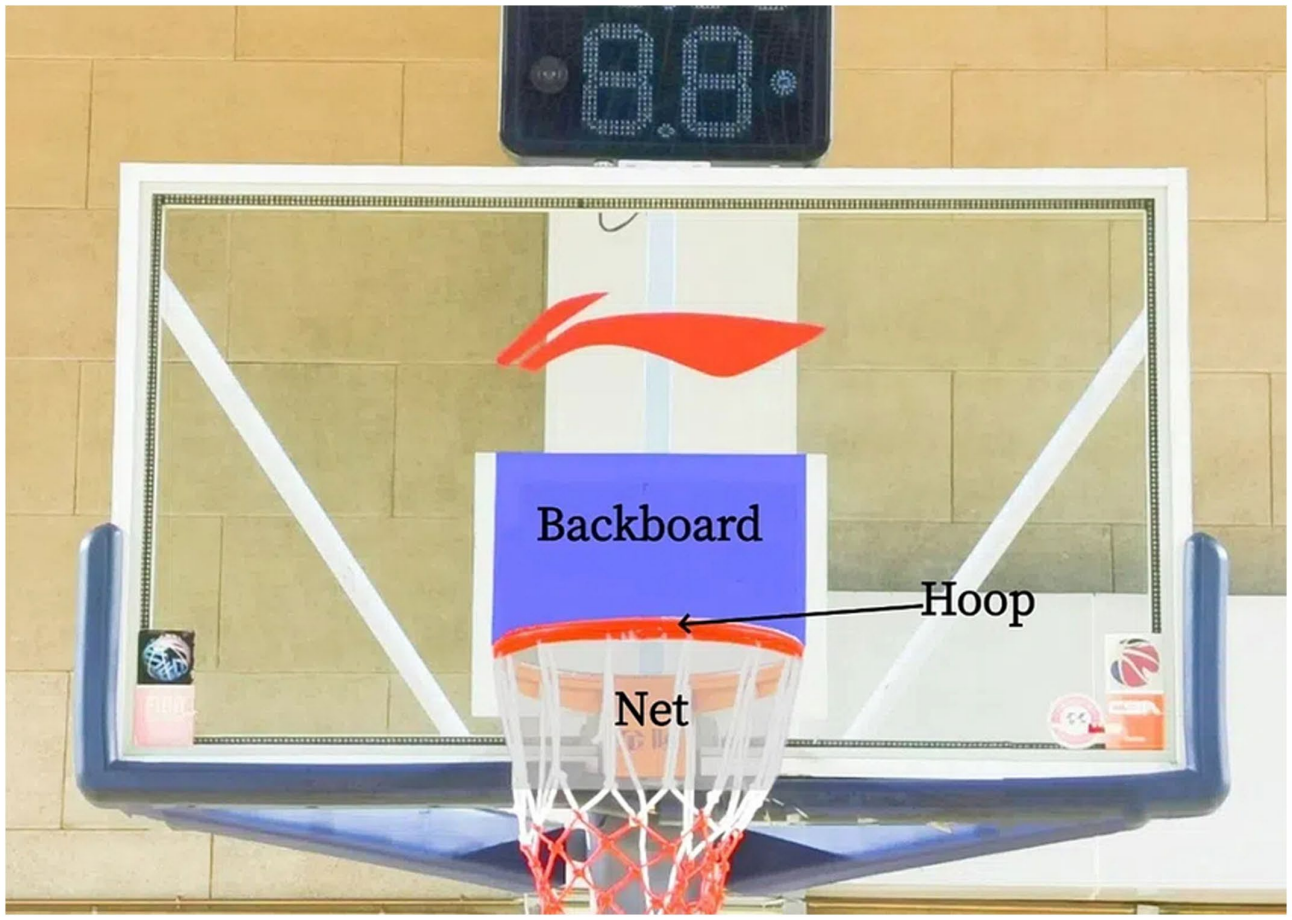
AOI based on shooting fixation position.

Three AOIs were defined objectively based on the basketball hoop structure using the eye-tracker’s scene camera coordinates: (1) Hoop: the metallic ring, (2) Backboard: the rectangular glass/board behind the hoop, and (3) Net: the net hanging from the hoop. Figure 1 illustrates the AOIs. These were validated through consultation with basketball experts and prior literature (Laby, 2020; Zhao et al., 2024b).

### Data analysis

Data were analyzed using SPSS 26.0. A series of 3 (Time) × 2 (Group) mixed ANOVAs were conducted for each dependent variable, as they represent theoretically linked components of visual attention. Normality was assessed using the Shapiro–Wilk test. Sphericity was evaluated with Mauchly’s test, with Greenhouse–Geisser corrections applied when violated. Partial eta-squared ( $\eta_{p}^{2}$ ) was reported as effect size. For significant interactions, Bonferroni-adjusted pairwise comparisons were conducted. Cohen’s *d* was calculated for pairwise comparisons. The significance level was set at *p* < 0.05.

## Results

Key findings are reported below; full statistical details are available in the tables. Descriptive statistics (Table 1) show the experimental group (EG) reduced the number of fixations across all AOIs and redistributed fixation duration toward the hoop, while the control group (CG) showed minimal changes. The EG’s mean 3PFG% increased from 0.43 to 0.48.

**Table 1:** Descriptive statistics (mean ± SD) for key measures across testing phases.

Variable	Group	Test phase	Hoop	Backboard	Net	Total number of fixations	Total fixation duration (ms)	3PFG%
Number of fixations	Experimental	Pre-test	3.2 ± 0.4	2.5 ± 0.3	2.1 ± 0.3	7.8 ± 0.6	–	–
		Mid-test	3.5 ± 0.5	2.0 ± 0.2	2.3 ± 0.3	7.8 ± 0.7	–	–
		Post-test	2.0 ± 0.3	1.4 ± 0.2	1.4 ± 0.2	4.8 ± 0.5	–	–
	Control	Pre-test	3.1 ± 0.4	2.4 ± 0.3	2.0 ± 0.3	7.5 ± 0.6	–	–
		Mid-test	3.0 ± 0.4	2.3 ± 0.3	1.9 ± 0.2	7.2 ± 0.6	–	–
		Post-test	2.8 ± 0.3	2.2 ± 0.2	1.7 ± 0.2	6.7 ± 0.5	–	–
Fixation duration (ms)	Experimental	Pre-test	1,012 ± 85	554 ± 45	579 ± 50	–	2,294 ± 120	–
		Mid-test	987 ± 80	634 ± 52	488 ± 42	–	2,243 ± 115	–
		Post-test	1,127 ± 90	453 ± 40	377 ± 35	–	2,081 ± 110	–
	Control	Pre-test	998 ± 82	560 ± 48	590 ± 52	–	2,302 ± 125	–
		Mid-test	1,005 ± 84	558 ± 46	585 ± 50	–	2,280 ± 118	–
		Post-test	986 ± 80	565 ± 47	590 ± 51	–	2,296 ± 120	–
3-Point FG%	Experimental	Pre-test	–	–	–	–	–	0.43 ± 0.03
		Mid-test	–	–	–	–	–	0.45 ± 0.04
		Post-test	–	–	–	–	–	0.48 ± 0.03

SD, Standard Deviation; 3PFG%, 3-Point Field Goal Percentage; ms, milliseconds.

Total Number of Fixations = Sum of number of fixations for Hoop, Backboard, and Net.

Total Fixation Duration = Sum of fixation durations for Hoop, Backboard, and Net.

Bold values indicate the post-test results for the Experimental group, which showed significant changes following the 8-week visual attention training intervention.

“–” denotes that the measure is not applicable for that specific row.

### Number of fixations

Mixed ANOVA results (Table 2) showed significant main effects of Time and Group, and significant Mixed ANOVA (Table 2) showed significant main effects of Time and Group, and significant Time × Group interactions for the hoop and backboard (all *p* < 0 0.05). For the net, the Time × Group interaction was significant (*p* = 0.022), but the main effect of Group was not (*p* = 0.102). *Post-hoc* tests (Table 3) confirmed that the EG significantly reduced fixations on the hoop (Pre: 3.2 vs. Post: 2.0, *p* < 0.001, *d* = 1.05), backboard (Pre: 2.5 vs. Post: 1.4, *p* = 0.001, *d* = 0.88), and net (Pre: 2.1 vs. Post: 1.4, *p* = 0.004, *d* = 0.72). The CG showed no significant changes (all *p* > 0.05, *d* < 0.25).

**Table 2:** Mixed ANOVA results for number of fixations.

Dependent variable	Effect	*F*	*p*	Partial *η*^2^ [95% CI]	Observed power
Hoop	Time	12.45	<0.001	0.37 [0.25, 0.48]	0.97
	Group	8.73	0.008	0.21 [0.09, 0.35]	0.81
	Time × Group	6.92	0.002	0.18 [0.07, 0.30]	0.75
Backboard	Time	9.84	<0.001	0.29 [0.17, 0.41]	0.89
	Group	5.12	0.035	0.13 [0.02, 0.26]	0.60
	Time × Group	5.01	0.011	0.14 [0.04, 0.26]	0.63
Net	Time	7.56	<0.001	0.24 [0.12, 0.36]	0.83
	Group	2.95	0.102	0.07 [0.00, 0.20]	0.38
	Time × Group	4.23	0.022	0.11 [0.02, 0.23]	0.53

**Table 3:** *Post hoc* comparisons for number of fixations.

Dependent variable	Comparison	Mean difference (ms)	*p*	Cohen’s *d* [95% CI]
Hoop	Exp. Post vs. Pre	−1.2 [−1.6, −0.8]	<0.001	1.05 [0.62, 1.48]
	Cont. Post vs. Pre	+0.3 [−0.1, 0.7]	0.148	0.25 [−0.08, 0.58]
Backboard	Exp. Post vs. Mid	−0.9 [−1.3, −0.5]	0.001	0.88 [0.46, 1.30]
	Cont. Mid vs. Pre	+0.1 [−0.3, 0.5]	0.612	0.10 [−0.23, 0.43]
Net	Exp. Post vs. Pre	−0.7 [−1.0, −0.4]	0.004	0.72 [0.31, 1.13]
	Cont. Post vs. Pre	+0.2 [−0.2, 0.6]	0.320	0.18 [−0.15, 0.51]

### Fixation duration

Mixed ANOVA (Table 4) showed significant main effects of Time and Group, and significant Time × Group interactions for all AOIs (all *p* < 0.01). *Post-hoc* tests (Table 5) revealed the EG increased fixation duration on the hoop (Pre: 1,012 ms vs. Post: 1,127 ms, *p* < 0.001, *d* = 1.24) and decreased duration on the backboard decreased duration on the backboard (Mid: 634 ms vs. Post: 453 ms, *p* < 0.001, *d* = 0.97) and net (Pre: 579 ms vs. Post: 377 ms, *p* < 0.001, *d* = 1.52). The control group showed no significant changes.

**Table 4:** Mixed ANOVA results for fixation duration.

Dependent variable	Effect	*F*	*p*	Partial *η*^2^ [95% CI]	Observed power
Hoop	Time	18.37	<0.001	0.42 [0.32, 0.51]	0.99
	Group	6.29	0.019	0.15 [0.04, 0.29]	0.67
	Time × Group	9.84	<0.001	0.31 [0.19, 0.43]	0.91
Backboard	Time	22.15	<0.001	0.49 [0.38, 0.58]	1.00
	Group	4.76	0.038	0.11 [0.02, 0.24]	0.56
	Time × Group	12.03	<0.001	0.36 [0.24, 0.48]	0.97
Net	Time	15.92	<0.001	0.38 [0.27, 0.47]	0.98
	Group	3.42	0.075	0.08 [0.00, 0.20]	0.43
	Time × Group	7.19	0.002	0.23 [0.11, 0.35]	0.82

**Table 5:** *Post-hoc* comparisons for fixation duration.

Dependent variable	Comparison	Mean Difference (ms)	*p*	Cohen’s *d* [95% CI]
Hoop	Exp. Post vs. Pre	+126 [98, 154]	<0.001	1.24 [0.81, 1.67]
	Cont. Post vs. Pre	−12 [−34, 10]	0.273	0.18 [−0.14, 0.50]
Backboard	Exp. Post vs. Mid	−89 [−112, −66]	<0.001	0.97 [0.55, 1.39]
	Cont. Mid vs. Pre	+5 [−18, 28]	0.651	0.06 [−0.26, 0.38]
Net	Exp. Post vs. Pre	−208 [−241, −175]	<0.001	1.52 [1.05, 1.99]
	Cont. Post vs. Pre	+34 [−5, 73]	0.089	0.31 [−0.05, 0.67]

### Fixation distribution

**Figure 2:**
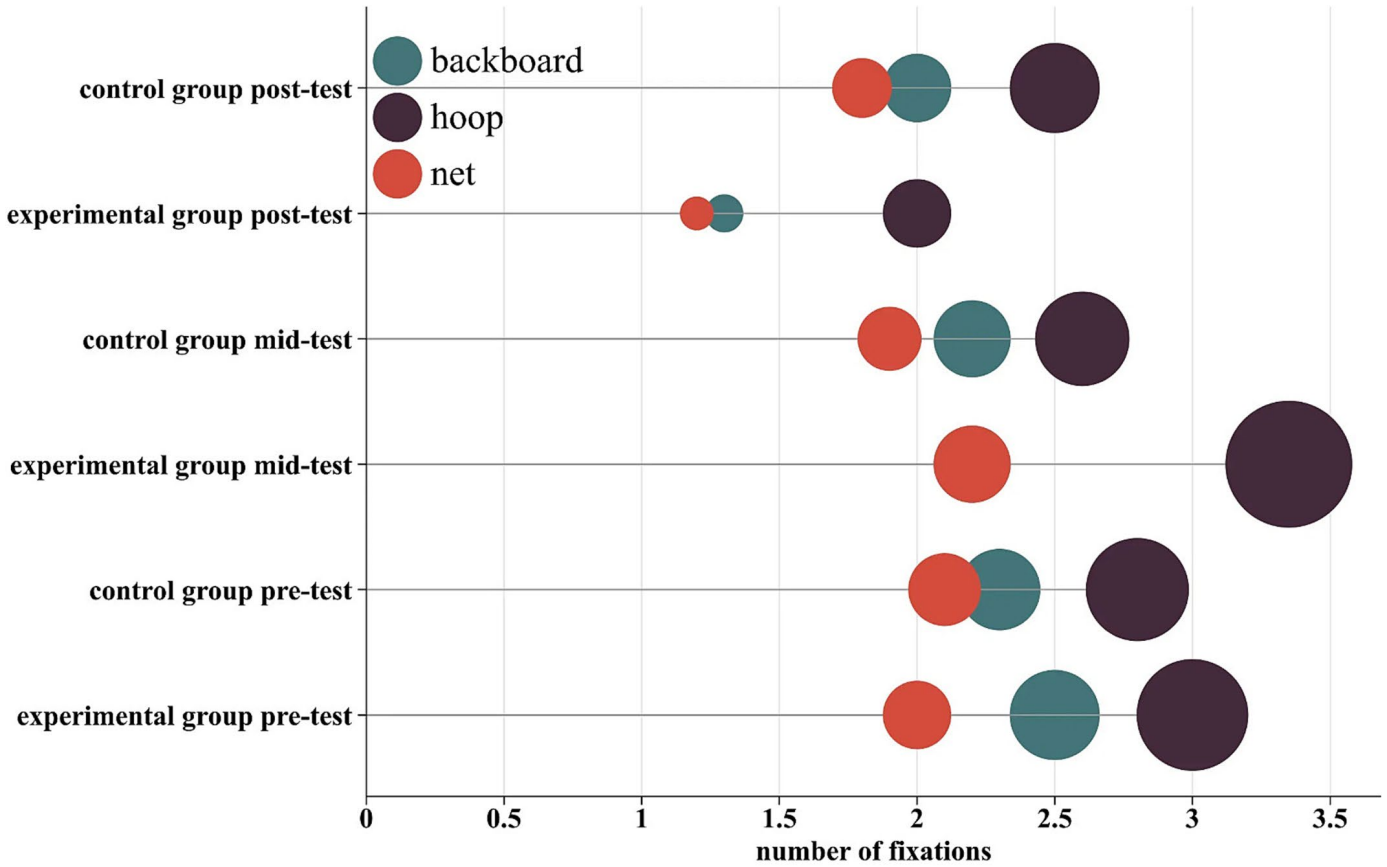
Distribution of the proportion of total fixations across areas of interest (AOI). The chart displays the proportion of total fixations falling within each AOI (Hoop, Backboard, Net) for the experimental and control groups at pre-test, mid-test, and post-test. This visualization complements the inferential statistics reported in Tables 1–3.

**Figure 3:**
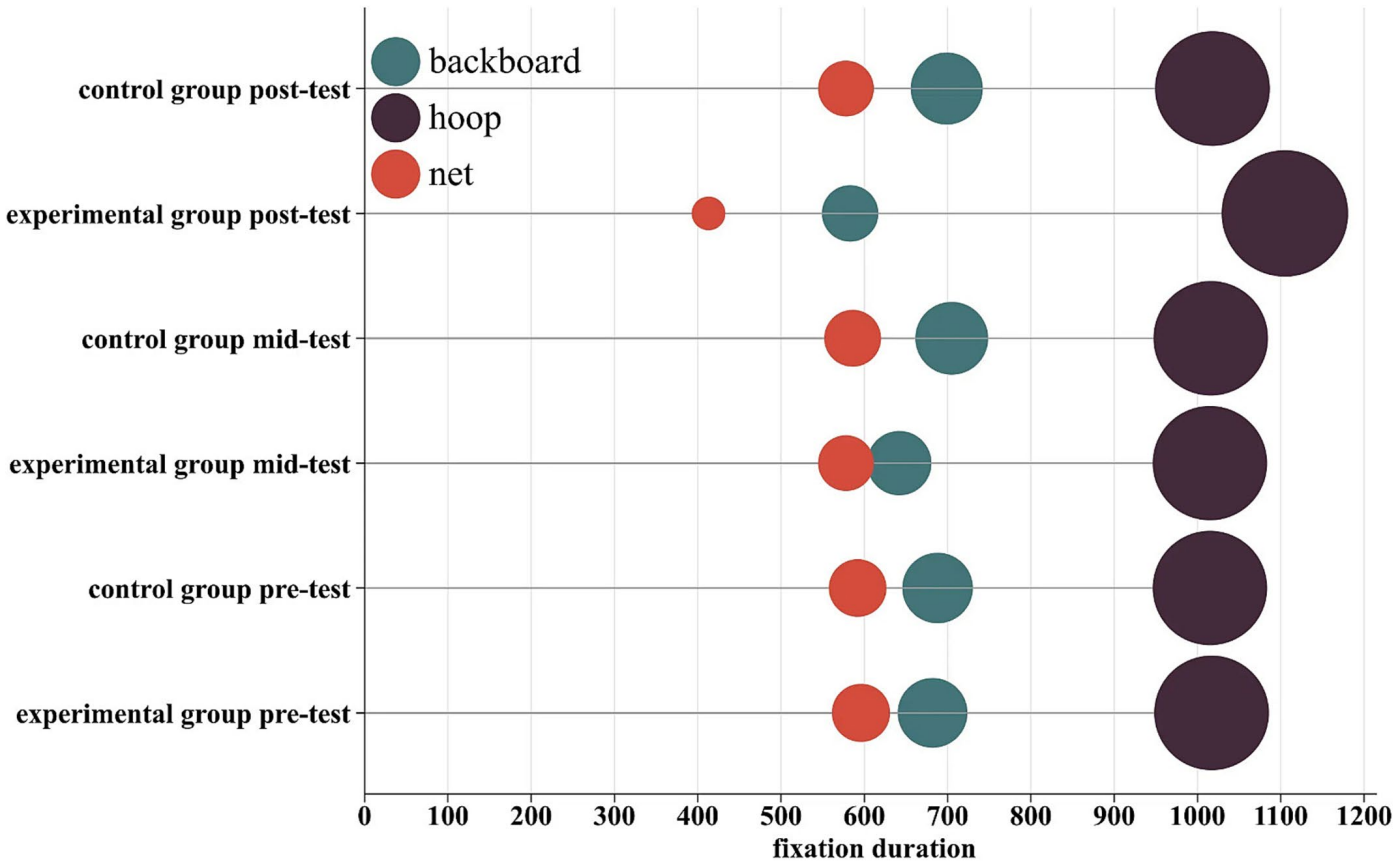
Distribution of the proportion of total fixation duration across areas of interest (AOI). The chart displays the proportion of total fixation duration spent within each AOI (Hoop, Backboard, Net) for the experimental and control groups at pre-test, mid-test, and post-test. This visualization complements the inferential statistics reported in Tables 1, 4 and 5.

To complement the inferential statistics, the overall distribution of visual attention across AOIs was examined descriptively (Figures 2, 3). The EG showed a progressive shift in number of fixation and fixation duration distribution toward the hoop from pre- to post-test. Specifically, the proportion of total fixations directed at the hoop increased from approximately 41% at pre-test to 48% at post-test, while the combined proportion for the backboard and net decreased from about 59 to 52%. Similarly, the proportion of total fixation duration spent on the hoop increased from roughly 44 to 54%, with a corresponding decrease for the backboard and net from 56 to 46%. In contrast, the CG’s distribution across AOIs remained relatively stable across all testing phases (hoop fixation proportion: ~41 to 42%; backboard and net: ~58 to 59%). These descriptive patterns of attentional redistribution align with and visually summarize the significant Time × Group interaction effects revealed by the mixed ANOVAs on individual AOI metrics (Tables 2, 3, 4, 5). They corroborate the finding that visual attention training effectively reshaped gaze patterns, leading to a more target-centric allocation of visual attention during 3-point shooting.

### 3-point field goal percentage

Mixed ANOVA (Table 6) revealed a significant main effect of Time (*F*_(2,36)_ = 15.73, *p* < 0.001, $\eta_{p}^{2}$ = 0.38) and Group (*F*_(1,18)_ = 9.24, *p* = 0.007, $\eta_{p}^{2}$ = 0.22), and a significant Time × Group interaction (*F*_(2,36)_ = 12.65, *p* < 0.001, $\eta_{p}^{2}$ = 0.29). *Post-hoc* comparisons (Table 7) *Post-hoc* tests showed the experimental group’s 3PFG% increased from 0.43 at pre-test to 0.48 at post-test (*p* < 0.001, *d* = 1.18), a 5.0% absolute improvement. The control group showed no change (*p* = 0.501, *d* = 0.12). The between-group difference at post-test was significant (+0.041, p < 0.001, *d* = 0.97).

**Table 6:** Mixed ANOVA results for 3-point field goal percentage (FG%).

Effect	*F*	*p*	Partial *η*^2^ [95% CI]	Observed power
Time	15.73	<0.001	0.38 [0.26, 0.48]	0.98
Group	9.24	0.007	0.22 [0.09, 0.36]	0.85
Time × Group	12.65	<0.001	0.29 [0.17, 0.41]	0.94
Time	15.73	<0.001	0.38 [0.26, 0.48]	0.98
Group	9.24	0.007	0.22 [0.09, 0.36]	0.85
Time × Group	12.65	<0.001	0.29 [0.17, 0.41]	0.94
Time	15.73	<0.001	0.38 [0.26, 0.48]	0.98
Group	9.24	0.007	0.22 [0.09, 0.36]	0.85
Time × Group	12.65	<0.001	0.29 [0.17, 0.41]	0.94

**Table 7:** *Post hoc* comparisons (Bonferroni-adjusted).

Comparison	Mean difference (ms)	*p*	Cohen’s *d* [95% CI]
Exp. Post vs. Pre	+0.046 [0.032, 0.060]	<0.001	1.18 [0.74, 1.62]
Cont. Post vs. Pre	+0.005 [−0.010, 0.020]	0.501	0.12 [−0.21, 0.45]
Exp. vs. Cont. (Post)	+0.041 [0.025, 0.057]	<0.001	0.97 [0.55, 1.39]

## Discussion

This study tested the effects of an 8-week theory-driven visual attention training protocol on the fixation behavior and shooting performance of semi-professional basketball players. The findings fully supported our hypotheses. The training successfully (1) optimized fixation behavior by reducing unnecessary fixations and reallocating processing time toward the primary target (hoop), and (2) led to a significant improvement in 3-point shooting accuracy.

The observed reduction in number of fixations across all AOIs and the specific increase in hoop fixation duration are consistent with the Quiet Eye framework and Attentional Focus Theory (Vickers, 1996; Moeinirad et al., 2020). The results suggest the training promoted a more efficient and stable external focus. The transient increase in fixations at mid-test may reflect a period of cognitive adjustment as players consciously attempted to alter ingrained habits, with consolidation into an expert-like profile occurring by post-test.

From a cognitive resource perspective (Heusler and Sutter, 2022), the optimized fixation behavior likely reflects enhanced attentional filtering and more efficient allocation of limited processing resources toward task-critical information (the hoop), while suppressing distractors (backboard/net). This improved information processing efficiency may have facilitated more automated motor programming (Laby, 2020; Jin et al., 2023), consistent with the Constrained Action Hypothesis (Matsumoto et al., 2022). The significant, though modest (5%), improvement in 3PFG% is practically meaningful at the semi-professional level, where small margins often determine competitive outcomes. It demonstrates that cognitive-perceptual training can yield measurable performance gains even in already-skilled athletes (Parr et al., 2019; Vickers et al., 2017).

This study extends Quiet Eye research by demonstrating that its key parameters are malleable through systematic training in a semi-professional population and that these changes are directly linked to performance improvement in a high-value task (3-point shooting).

### Limitations and future research

Several limitations must be acknowledged. First, the sample size was adequate for detecting medium-to-large effects but was relatively small and comprised exclusively of elite female university players, limiting generalizability. Second, the lack of blinding (participants and coaches were aware of group assignment) may have introduced expectancy effects. Third, while the 8-week dose was effective, the long-term retention of these benefits and the optimal training dosage remain unknown. Fourth, the control group received general coaching feedback, constituting an active control condition but not a placebo control.

Future research should employ larger, more diverse samples and include longer-term follow-ups (e.g., 3–6 months) to assess sustainability. Blinded assessors and placebo control conditions (e.g., sham vision training) would strengthen the design. Investigating the underlying neurocognitive mechanisms (e.g., via EEG) and developing individualized training protocols are promising directions.

## Conclusion

This study provides evidence that an 8-week visual attention training protocol can effectively modify fixation behavior and improve 3-point shooting accuracy in semi-professional basketball players. The training promoted a more efficient and stable visual focus on the target, underscoring the value of integrating cognitive-perceptual training into sport-specific skill development.

## Data Availability

The original contributions presented in the study are included in the article/supplementary material, further inquiries can be directed to the corresponding author.
